# Clinical and Molecular Delineation of *KAT6B*‐Related Disorders: Novel Variants and Refined Genotype–Phenotype Correlations

**DOI:** 10.1155/humu/6378376

**Published:** 2026-09-24

**Authors:** Vito Luigi Colona, Maria Gnazzo, Annalidia Donato, Lavinia Fioretti, Alessandra Terracciano, Delia Monopoli, Paola Sabrina Buonuomo, Michaela Veronika Gonfiantini, Donatella Lettori, Roberta Taurisano, Tommaso Mazza, Daniela Concolino, Mafalda Mucciolo, Marina Macchiaiolo, Antonio Novelli, Davide Vecchio

**Affiliations:** ^1^ Rare Diseases and Medical Genetics Unit, Bambino Gesù Children′s Hospital, IRCCS, Rome, Italy, istitutotumori.na.it; ^2^ Unit of Neurorehabilitation, Bambino Gesù Children′s Hospital, IRCCS, Rome, Italy, istitutotumori.na.it; ^3^ Laboratory of Medical Genetics, Translational Cytogenomics Research Unit, Bambino Gesù Children′s Hospital, IRCCS, Rome, Italy, istitutotumori.na.it; ^4^ Computational Biology and Bioinformatics Unit, Agostino Gemelli University Hospital Foundation, IRCCS, Rome, Italy, istitutotumori.na.it; ^5^ Pediatric Unit, Department of Medical and Surgical Sciences, Magna Graecia University of Catanzaro, Catanzaro, Italy, unicz.it

**Keywords:** Genitopatellar syndrome, genotype–phenotype correlation, *KAT6B*-related disorders, obstructive sleep apnea, RNA sequencing, Say–Barber–Biesecker–Young–Simpson syndrome

## Abstract

KAT6B‐related disorders (KRDs) comprise a *spectrum* of developmental disorders ranging from Genitopatellar syndrome (GPS) to Say–Barber–Biesecker–Young–Simpson syndrome (SBBYSS), with increasing recognition of intermediate phenotypes. Although genotype–phenotype correlations have progressively emerged, the molecular basis of phenotypic variability remains incompletely understood. We performed comprehensive clinical phenotyping and trio‐based whole‐exome sequencing (WES) in two unrelated individuals carrying novel de novo *KAT6B* variants. Transcriptomic analysis was undertaken for the noncanonical intronic variant using peripheral blood‐derived RNA. An updated review of molecularly confirmed KRDs was also performed to reassess genotype–phenotype correlations and protein domain‐specific variant distribution. Patient 1 carried a novel nonsense variant (c.5347G>T, p.Glu1783Ter) and presented with a predominantly SBBYSS‐like phenotype. Patient 2 harbored a novel noncanonical intronic variant (c.3665‐21C>A) associated with a severe multisystem phenotype overlapping the GPS *spectrum*. RNA sequencing demonstrated preserved canonical exon 17–18 splicing but an approximately 50% reduction in *KAT6B* transcript abundance, supporting a dosage‐sensitive pathogenic mechanism. Review of 203 published individuals identified 133 distinct pathogenic variants, confirmed exon 18 as the major mutational hotspot, and suggested domain‐specific genotype–phenotype trends, including enrichment of SBBYSS‐associated phenotypes among variants affecting the histone acetyltransferase (HAT) domain. Both patients also exhibited severe upper airway dysfunction with obstructive sleep apnea (OSA). These findings expand the molecular and phenotypic *spectrum* of KRDs, provide supportive transcriptomic evidence for a potential functional intronic variant, refine current genotype–phenotype correlations, and highlight upper airway dysfunction as a clinically relevant but likely underrecognized manifestation across the KRD *spectrum*.

## 1. Introduction

*KAT6B* (lysine acetyltransferase 6B; previously known as *MYST4* or MORF‐related gene; MIM ∗605880) encodes a member of the MYST family of histone acetyltransferases (HATs), a highly conserved group of epigenetic regulators involved in chromatin remodeling and transcriptional control during embryogenesis [1–4]. Located on chromosome 10q22.2, *KAT6B* (NM_012330.4) encodes a 2,073‐amino‐acid multidomain protein comprising the N‐terminal NEMM domain, tandem plant homeodomain (PHD) zinc‐finger motifs, the catalytic MYST HAT domain, an acidic region, and a C‐terminal serine/methionine‐rich (SM) transcriptional regulatory domain [3]. These domains coordinate chromatin recognition, protein–protein interactions, transcriptional complex assembly, and HAT activity, thereby regulating gene‐expression programs essential for tissue specification and organogenesis [1–3]. Consistent with these functions, experimental studies have demonstrated critical roles for *KAT6B* in skeletal and craniofacial morphogenesis, central nervous system (CNS) patterning, endocrine differentiation, and genitourinary development [5, 6]. More recently, functional studies have further implicated *KAT6B* in neural progenitor differentiation, neuronal maturation, and transcriptional networks involved in cortical development, providing a mechanistic basis for the frequent occurrence of structural brain abnormalities, neurodevelopmental impairment, and epilepsy observed in affected individuals [7, 8].

The clinical relevance of *KAT6B* emerged following the identification of heterozygous pathogenic variants underlying two previously recognized developmental syndromes, namely Genitopatellar syndrome (GPS) (OMIM #606170) and Say–Barber–Biesecker–Young–Simpson syndrome (SBBYSS) (OMIM #603736), also known as Ohdo syndrome, SBBYS variant [3, 4, 9]. Collectively referred to as KAT6B‐related disorders (KRDs), these autosomal dominant conditions are usually caused by de novo variants. GPS is typically characterized by agenesis or hypoplasia of the patellae, genital anomalies, agenesis of the corpus callosum (CC), renal malformations, flexion contractures, and severe neurodevelopmental impairment, whereas SBBYSS more commonly presents with characteristic craniofacial dysmorphism, ptosis, lacrimal abnormalities, cleft palate, hearing impairment, congenital hypothyroidism, and genital defects [3, 4]. However, the growing number of molecularly confirmed cases has progressively challenged this dichotomous classification, revealing substantial phenotypic overlap and marked interindividual variability. Increasing evidence now supports the concept that GPS and SBBYSS represent the opposite ends of a continuous phenotypic *spectrum* rather than discrete nosological entities [6, 10].

To date, most pathogenic *KAT6B* variants are truncating alterations, including nonsense and frameshift variants, which predominantly cluster within the distal portion of the gene, particularly exon 18, although splice‐altering and, less frequently, missense variants have also been reported [10]. Emerging genotype–phenotype correlations indicate that both variant position and predicted molecular consequence contribute to clinical variability. More recently, the affected protein domain has also been proposed as an additional determinant of phenotypic expression, although domain‐specific genotype–phenotype relationships remain incompletely defined because of the limited number of functionally characterized cases [6, 10]. Multiple pathogenic mechanisms have been proposed, including truncated proteins escaping nonsense‐mediated decay (NMD), dominant‐negative or altered transcriptional effects, and dosage‐sensitive haploinsufficiency, which are not necessarily mutually exclusive [4, 6, 10]. In contrast, functional evidence supporting the pathogenicity of noncanonical splice‐region variants remains scarce, and their interpretation frequently requires transcript‐level analyses beyond conventional in silico prediction.

Beyond the core syndromic manifestations, the clinical *spectrum* associated with KRDs appears considerably broader than originally recognized. Airway abnormalities, obstructive sleep apnea (OSA), feeding and swallowing dysfunction, endocrine disorders, renal involvement, and complex neuroradiological abnormalities are increasingly reported, although many of these features likely remain underrecognized because of limited longitudinal follow‐up and incomplete multidisciplinary characterization. In particular, upper airway dysfunction related to craniofacial abnormalities, glossoptosis, or altered pharyngeal dynamics may represent a clinically relevant but insufficiently investigated contributor to morbidity, requiring early recognition and specialized management. Likewise, the natural history of neurological manifestations, including structural brain abnormalities, epilepsy, and neurodevelopmental trajectories, remains incompletely defined, particularly in individuals with overlapping GPS‐SBBYSS phenotypes [11].

Here, we describe two unrelated individuals harboring previously undescribed de novo *KAT6B* variants, including a novel noncanonical splice‐region variant investigated by RNA sequencing. By combining comprehensive multidisciplinary phenotyping, transcriptomic functional characterization, and an updated review of published molecularly confirmed KRDs, we further expand the molecular and clinical *spectrum* of KRDs and refine current genotype–phenotype correlations, with particular emphasis on domain‐specific variant distribution and the interpretation of noncanonical splice‐region variants.

## 2. Materials and Methods

### 2.1. Clinical Evaluation

Both patients underwent comprehensive multidisciplinary evaluation at Bambino Gesù Children′s Hospital, IRCCS, Rome, Italy. Clinical data were retrospectively collected from medical records and included prenatal history, neonatal course, neurological, neuroradiological, craniofacial, otolaryngological, respiratory, endocrinological, gastrointestinal, orthopedic, cardiological, nephro‐urological, and ophthalmological assessments. Ancillary investigations were reviewed when available, including brain magnetic resonance imaging (MRI), electroencephalography (EEG), polysomnography, airway endoscopy, videofluoroscopic swallowing studies, echocardiography, and audiological examinations. Clinical features were systematically compared with previously reported *KAT6B*‐related phenotypes.

### 2.2. Genetic Analyses

Genomic DNA was extracted from peripheral blood samples obtained from the probands and their parents. Whole‐exome sequencing (WES) libraries were prepared using either the Twist Human Comprehensive Exome Kit (Twist Bioscience, San Francisco, California, United States) or the KAPA HyperExome Probes V2 kit (Roche, Basel, Switzerland), according to the manufacturers′ protocols. Paired‐end sequencing was performed on an Illumina NovaSeq 6000 platform (Illumina, San Diego, California, United States).

Sequence reads were aligned to the GRCh37/hg19 human reference genome. Variant calling was performed using the DRAGEN Enrichment Pipeline v4.0.3 (Illumina), and variant annotation was conducted using the Geneyx Analysis Suite (LifeMap Sciences, Tel Aviv, Israel). Variants were prioritized through phenotype‐driven analysis and filtered according to sequencing quality, predicted molecular consequence, compatibility with the expected inheritance model, and rarity in population databases, including gnomAD v2.1.1. For the dominant de novo model, variants absent or rare (minor allele frequency [MAF] ≤ 0.1*%*) in population databases were prioritized.

Variants were classified according to the American College of Medical Genetics and Genomics and the Association for Molecular Pathology (ACMG/AMP) standards for sequence‐variant interpretation, incorporating subsequent ClinGen Sequence Variant Interpretation recommendations where applicable [12]. Whenever available, variant interpretation was further supported by segregation analysis, detailed phenotypic correlation, and functional evidence.

### 2.3. In Silico Variant Interpretation

The potential functional impact of identified variants was assessed using multiple computational prediction tools. Predicted deleteriousness was evaluated using Combined Annotation Dependent Depletion (CADD) (v1.6), Rare Exome Variant Ensemble Learner (REVEL), and SpliceAI. Evolutionary conservation was assessed using phyloP 100‐way vertebrate scores. Regional tolerance to missense variation was explored using MetaDome [13] and was interpreted descriptively rather than as independent evidence of pathogenicity. Whenever appropriate, predictions were interpreted in the context of protein‐domain organization and currently available genotype–phenotype correlations reported for KRDs.

### 2.4. RNA Sequencing and Transcript Analysis

Transcriptomic analysis was performed on RNA extracted from peripheral blood samples. RNA sequencing data were analyzed to investigate potential splicing abnormalities and to compare overall *KAT6B* transcript abundance between samples. Paired‐end reads from the proband and the unaffected mother were quality‐filtered and aligned to the GRCh38 human reference genome using STAR (v2.7) with the NCBI RefSeq‐based GRCh38.p13 annotation. Two‐pass mapping (‐‐twopassMode Basic) was enabled to improve splice‐junction detection. Per‐base read depth across the *KAT6B* locus (chr10: 74,826,000–75,033,000, GRCh38) was extracted with samtools (v1.17). Splice junctions were quantified from the STAR SJ.out. tab output, and usage of the canonical exon 17–exon 18 junction (intron chr10: 75,025,250–75,028,488, GRCh38; transcript NM_012330.4) was compared between the two samples. To investigate potential mis‐splicing associated with c.3665‐21C>A, STAR junction output and read alignments were inspected for cryptic donor or acceptor junctions, intron retention, and exon‐skipping events in the region surrounding the variant, including a ± 250‐bp interval. Expression levels of housekeeping genes *ACTB* and *GAPDH* were used as internal controls to exclude technical bias and global differences in sequencing depth. Relative *KAT6B* transcript abundance was estimated from *locus* read coverage, normalized to the housekeeping gene expression, and expressed as a mother‐to‐proband ratio.

### 2.5. Literature Review

To contextualize the molecular and clinical findings observed in the present study, a comprehensive review of the published literature was performed to identify individuals with molecularly confirmed KRDs. The PubMed and Scopus databases were searched up to June 2026 using combinations of the terms “*KAT6B*,” “Genitopatellar syndrome,” “Say‐Barber‐Biesecker‐Young‐Simpson syndrome,” “SBBYSS,” and “GPS.” Additional relevant publications were identified through manual screening of the reference lists of retrieved articles. Only English‐language reports with full‐text availability and sufficient molecular information to define the underlying *KAT6B* variant were included. Duplicate reports, conference abstracts, and publications lacking molecular characterization were excluded. Clinical and molecular data were extracted to generate the datasets summarized in Table S1. Whenever sufficient clinical information was available, reported individuals were categorized as presenting with SBBYSS, GPS, or an intermediate *KAT6B*‐related phenotype according to the original clinical descriptions. Variants were further classified according to their genomic localization, predicted molecular consequence, and corresponding protein domains based on the canonical *KAT6B* protein sequence (UniProt accession Q8WYB5).

### 2.6. Ethics Statement

Genetic investigations were conducted in accordance with the principles of the Declaration of Helsinki. Written informed consent for genetic investigations and publication of anonymized clinical data and images was obtained from the parents or legal guardians of both patients. The study was approved by the local Institutional Review Board (IRB) of Bambino Gesù Children′s Hospital, IRCCS, Rome, Italy (n. RAP‐2026‐0002).

## 3. Results

### 3.1. Patient 1

Patient 1 was a 4‐month‐old male infant referred to our center for multidisciplinary evaluation because of a complex polymalformative phenotype involving multiple congenital anomalies. The main clinical findings are summarized in Table 1. He was the only child of healthy nonconsanguineous parents and was born at 38 weeks and 2 days of gestation following an uncomplicated pregnancy except for prenatal ultrasound findings of polyhydramnios and small‐for‐gestational‐age (SGA) growth parameters.

**Table 1:** Genotype–phenotype correlation of the two *KAT6B* patients diagnosed at our Hospital.

		Patient 1	Patient 2
Genotype (inheritance)		Het (de novo)	Het (de novo)
cDNA (NM_012330.4)		c.5347G>T	c.3665‐21C>A
Position		Exon 18	Intron 17
Protein (NP_036462.2)		p.Glu1783Ter	p.?
Variant type		Nonsense	Splicing
ACMG		C5	C4
Gender (M/F)		M	F
Age at last evaluation		4 months	4 years and 6 months
Phenotype		SBBYSS‐like	GPS
Neurological features	ID/DD	N.A.	+
	Hypotonia	+	−
	Seizures	−	+
	Microcephaly	+	−
	CC anomalies	+ (thinning)	+ (agenesis)
	Periventricular neuronal heterotopia	−	−
	Cerebellar involvement	N.A.	+
Musculoskeletal features	Joint contractures	−	+
	Patellar anomalies	−	+
	Long thumbs or long great toes	−	−
Congenital heart defect (type)		+ (PFO)	+ (ASD)
Craniofacial features	Mask‐like facies	−	+
	Blepharophimosis	−	+
	Ptosis	−	+
	Lacrymal duct anomalies	−	−
	Palate anomalies	+	+
	Dental anomalies	−	+
GI and feeding issues	Feeding difficulties	+	+
	Anal anomalies	−	+
Tracheomalacia		−	−
Laryngomalacia		−	+
Respiratory distress		+	+
Thyroid anomalies		+	+
Renal and urinary tract anomalies		+	+
Genital anomalies		Bifid scrotum cryptorchidism	Ambiguous genitalia clitoral hypertrophy
Hearing impairment and EAC malformations		+	+
Optic atrophy or hypoplasia or cortical visual impairment		+	−

Abbreviations: −, absent; +, present; ACMG, American College of Medical Genetics and Genomics; C4, Class 4 (likely pathogenic variant); C5, Class 5 (pathogenic variant); CC, corpus callosum; DD, developmental delay; EAC, external auditory canal; F, female; GI, gastrointestinal; GPS, Genitopatellar syndrome; Het, heterozygous; ID, intellectual disability; M, male; N.A., not applicable; SBBYSS, Say–Barber–Biesecker–Young–Simpson syndrome.

Neonatal adaptation was complicated by respiratory distress requiring brief positive‐pressure ventilation followed by transient noninvasive respiratory support. Persistent oxygen desaturations during feeding were observed during the neonatal period. Physical examination demonstrated hypoplastic bifid *scrotum* with bilateral cryptorchidism, secondary cleft palate, bilateral II–III toe syndactyly, micrognathia, narrow thorax, bilateral preauricular pits with blind endings, and generalized hypotonia.

Endocrinological investigations demonstrated congenital hypothyroidism with elevated thyroid‐stimulating hormone (TSH) levels, prompting initiation of levothyroxine replacement therapy. Thyroid ultrasound excluded structural abnormalities. Ophthalmological evaluation showed mild optic nerve hypoplasia, whereas audiological assessment confirmed mild bilateral conductive hearing loss following abnormal newborn hearing screening.

**Figure 1:**
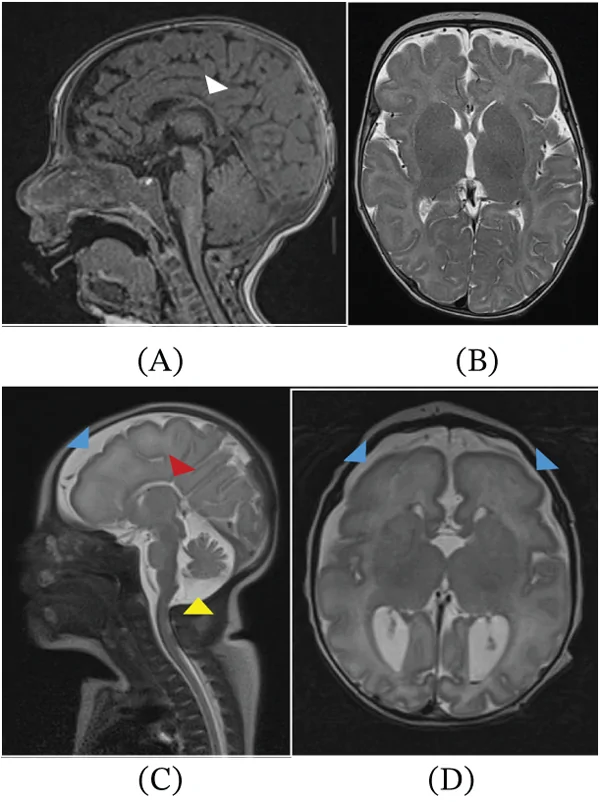
Brain magnetic resonance imaging (MRI) findings in the two patients with *KAT6B*‐related disorders. Patient 1: (A) sagittal T1‐weighted and (B) axial T2‐weighted images demonstrating thinning of the corpus callosum (white arrow). Patient 2: (C) sagittal and (D) axial T2‐weighted images showing complete agenesis of the corpus callosum (red arrow), marked gyral simplification involving the frontal and anterior temporal lobes (light blue arrows), and cerebellar vermian hypoplasia (yellow arrow).

Brain ultrasonography demonstrated a small right choroid plexus cyst. Brain MRI, performed at 4 months of age, revealed thinning of the CC without additional supratentorial or infratentorial structural abnormalities (Figure 1A,B). Neurological examination showed mildly reduced variability of spontaneous movements, mild generalized hypertonia, and preserved interaction with the environment.

Respiratory assessment disclosed severe upper airway obstruction. Overnight pulse oximetry with capnography demonstrated severe OSA. Flexible airway endoscopy revealed retroglossoptosis with dynamic worsening during sleep, resulting in partial obstruction of the oro‐ and hypopharyngeal airway and secondary collapse of the epiglottis over the laryngeal vestibule. Respiratory support was progressively escalated from high‐flow nasal cannula therapy to continuous positive airway pressure (CPAP), resulting in marked clinical improvement.

Videofluoroscopic swallowing study demonstrated rapid swallowing, delayed swallow reflex, velopharyngeal insufficiency, nasal regurgitation, and aspiration of thin liquids, whereas swallowing of thickened feeds remained safe.

Cardiological assessment revealed a patent foramen ovale (PFO) and mild dilation of the aortic annulus. Renal ultrasound demonstrated borderline reduced cortico‐medullary differentiation with mild renal asymmetry but no major structural abnormalities. Metabolic screening and abdominal investigations were otherwise unremarkable.

### 3.2. Patient 2

Patient 2 was a 4‐year‐and‐6‐month‐old girl referred for multidisciplinary evaluation because of a severe multisystem developmental disorder involving the CNS, upper airway, musculoskeletal system, gastrointestinal tract, endocrine organs, and genitourinary system. The principal clinical findings are summarized in Table 1.

Prenatal investigations had already suggested an underlying developmental syndrome, revealing agenesis of the CC and cavum septi pellucidi, reduced gastric filling, umbilical vein ectasia, bilateral hydronephrosis, and bilateral clubfoot. Pregnancy was further complicated by premature rupture of membranes at 31 weeks of gestation, followed by spontaneous vaginal delivery at 36 weeks. Birth weight was 2490 g.

Immediately after birth, the patient developed respiratory distress associated with choanal stenosis, requiring brief intubation followed by noninvasive ventilatory support. Additional congenital anomalies included ambiguous genitalia and anorectal malformation. Severe nasal obstruction required early endoscopic correction of nasal synechiae and choanal stenosis, followed by revision procedures that ultimately restored satisfactory airway patency.

Longitudinal neurological follow‐up demonstrated profound global developmental delay and severe intellectual disability. Brain MRI, performed during the neonatal period (1 month of age), confirmed complete agenesis of the CC, marked gyral simplification involving the frontal and anterior temporal lobes, and reduced hippocampal inversion (Figure 1C,D). At 3 years of age, the patient developed generalized epileptic seizures. EEG demonstrated diffuse background disorganization with multifocal epileptiform abnormalities, leading to initiation of levetiracetam therapy.

Respiratory evaluation documented persistent upper airway dysfunction with moderate‐to‐severe OSA requiring nocturnal noninvasive ventilation. Endoscopic airway examination demonstrated posterior displacement of the tongue base with dynamic collapse of the epiglottis over the laryngeal vestibule during sleep. Audiological assessment revealed severe bilateral mixed hearing loss requiring hearing amplification.

Gastrointestinal involvement included severe feeding difficulties, dysphagia, gastroesophageal reflux disease, and failure to thrive, requiring Nissen fundoplication followed by placement of a percutaneous endoscopic gastrostomy (PEG). Endocrinological investigations documented congenital hypothyroidism requiring levothyroxine replacement therapy together with recurrent episodes of hyperinsulinemic hypoglycemia that remain under investigation.

Genitourinary abnormalities comprised anteriorly displaced *anus*, clitoral hypertrophy, bicornuate *uterus*, bilateral renal hyperechogenicity with reduced cortico‐medullary differentiation, hydronephrosis, nephrolithiasis, and recurrent urinary tract infections.

Orthopedic manifestations included bilateral clubfoot with tendon contractures, bilateral neurogenic knee flexion deformities requiring surgical correction, and bilateral patellar agenesis.

Cardiological investigations demonstrated two ostium secundum atrial septal defects associated with moderate left‐to‐right shunting and transient dilation of the right cardiac chambers. Follow‐up echocardiography showed normalization of right ventricular dimensions, whereas mild dilation of the ascending aorta persisted. Chest computed tomography (CT) additionally demonstrated asymmetric lung volumes with mild mediastinal shift and focal ground‐glass opacities involving the right upper lobe.

### 3.3. Molecular Findings

Trio‐based WES identified two previously unreported de novo *KAT6B* variants in the two unrelated patients.

In Patient 1, a heterozygous nonsense variant, c.5347G>T (p.Glu1783Ter), was identified within exon 18 and classified as pathogenic (Class 5) according to ACMG/AMP criteria (PM2 moderate; PS2 and PVS1 strong). The variant is predicted to introduce a premature termination codon within the distal portion of the coding sequence, consistent with previously established pathogenic mechanisms underlying KRDs.

In Patient 2, WES identified a heterozygous intronic variant, c.3665‐21C>A, located within intron 17 outside the canonical splice acceptor sequence. The variant was absent from population databases and was classified as likely pathogenic (Class 4) according to ACMG/AMP criteria (PM2 moderate; PS2 strong). Owing to its location within a noncanonical splice region, transcriptomic analyses were undertaken to investigate its functional consequences.

RNA sequencing performed on peripheral blood‐derived RNA demonstrated preservation of the canonical exon 17–exon 18 splice junction in both the proband and the unaffected mother, with no evidence of exon skipping, cryptic splice‐site activation, or abnormal exon 17–exon 18 junction usage. Consistent with these findings, per‐base read depth across exons 16–18 was comparable in shape between the proband and the unaffected mother, with no selective loss of exon 18 coverage in the proband. Nevertheless, quantitative transcript analysis demonstrated an approximately two‐fold reduction in overall *KAT6B* transcript abundance in the proband compared with the mother (mother/proband ratio ≈ 2.1) (Figure S1A,B). In contrast, the housekeeping genes *ACTB* and *GAPDH* exhibited comparable expression levels between samples, excluding relevant technical bias or global transcriptional differences. The reduction therefore appeared to be gene specific (Figure S1B).

Although the precise molecular mechanism could not be directly established, the observed transcriptomic profile is compatible with preferential degradation of transcripts originating from the mutant allele, resulting in predominant expression of the wild‐type transcript. Collectively, these findings provide functional evidence supporting the pathogenicity of the c.3665‐21C>A variant and indicate that its pathogenic effect is unlikely to result from detectable disruption of canonical splicing in peripheral blood. Rather, the observed reduction in transcript abundance supports a dosage‐sensitive mechanism compatible with *KAT6B* haploinsufficiency.

## 4. Discussion

KRDs comprise a clinically heterogeneous group of developmental conditions spanning a broad phenotypic *continuum* between the classical manifestations of GPS and SBBYSS. Increasing recognition of overlapping and intermediate phenotypes has progressively challenged the traditional distinction between these syndromes, supporting a *spectrum* model in which clinical expression reflects the combined effects of variant location, molecular consequence, residual protein function, and additional genetic or epigenetic modifiers [5, 6, 14–16]. The two individuals described here exemplify this *continuum*: Patient 1 showed a predominantly SBBYSS‐like phenotype, although he lacked the major clinical features classically associated with SBBYSS and instead displayed several findings overlapping with the GPS *spectrum*, most notably genital anomalies; Patient 2 showed a predominantly GPS phenotype, characterized by multiple major GPS‐associated features, while also exhibiting craniofacial findings commonly reported in SBBYSS (Table 1). Nevertheless, both shared several clinically relevant manifestations, including upper airway dysfunction, OSA, feeding difficulties, hearing impairment, and endocrine abnormalities, highlighting the substantial overlap that exists across the KRD *spectrum*.

*KAT6B* encodes a lysine acetyltransferase comprising several functional domains (i.e., NEMM, PHDs, HAT, acid region, and SM) [3]. Through acetylation of histone H3 and regulation of chromatin accessibility, *KAT6B* controls transcriptional programs essential for embryonic patterning, skeletal development, craniofacial morphogenesis, neurogenesis, endocrine differentiation, and genitourinary development [8]. In this context, KRDs can also be considered as part of the broader group of chromatinopathies, a clinically heterogeneous class of Mendelian developmental disorders caused by pathogenic variants in genes encoding regulators of the epigenetic machinery [17, 18]. Consistent with this framework, experimental studies have demonstrated that disruption of *KAT6B* function alters neural progenitor cell differentiation and affects the expression of developmental regulators, including *SOX2,* as well as other neurodevelopmentally relevant pathways, providing a mechanistic framework for the frequent occurrence of structural brain abnormalities, intellectual disability, and epilepsy observed across the clinical *spectrum* of KRDs. Importantly, *KAT6B* also functions as a scaffold for multiprotein chromatin‐remodeling complexes, suggesting that variants affecting distinct structural domains may differentially perturb transcriptional regulatory networks beyond catalytic acetyltransferase activity alone [6–8].

To better contextualize our findings within the current molecular landscape of KRDs, we reviewed all molecularly confirmed cases for which individual genetic data were available. Across 203 published individuals, we identified 133 distinct variants, including three large deletions involving the *KAT6B locus*. Truncating variants represented most disease‐causing alleles, with frameshift variants accounting for 58.6% of reported alterations and nonsense variants for 29.3%. In contrast, missense variants were uncommon (5.3%), whereas splice‐site variants represented only 3.8% of all reported molecular defects. This distribution strongly supports disruption of normal *KAT6B* dosage or function as the predominant pathogenic mechanism underlying the disorder (Table S1).

Variant distribution across the gene was markedly nonrandom. Exon 18 emerged as the principal mutational hotspot, harboring 81 of the 133 distinct variants identified in the literature (60.9%) (Table S1). This observation confirms previous reports implicating the distal portion of the gene as the region most frequently involved in disease pathogenesis [6, 10]. However, our analysis further suggests that genotype–phenotype correlations are more complex than a simple exon‐based classification. Variants associated with GPS preferentially clustered around the distal portion of exon 17 and the proximal region of exon 18, consistent with previous observations identifying this interval as the genomic region most strongly associated with the GPS phenotype [6, 10]. Conversely, SBBYSS‐associated variants were distributed across multiple exons and domains, without evidence of a single discrete hotspot. Intermediate phenotypes showed extensive overlap with both groups, reinforcing the concept that GPS and SBBYSS represent phenotypic extremes of a continuous developmental *spectrum* rather than discrete molecular entities (Table S1). Overall, these observations indicate that variant position alone is insufficient to predict clinical outcome, reinforcing the contribution of additional molecular mechanisms.

**Figure 2:**
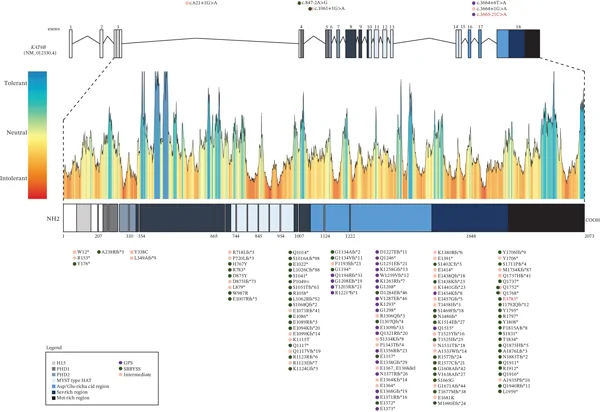
MetaDome tolerance landscape and distribution of reported *KAT6B* variants across the gene and protein domains. Schematic representation of the *KAT6B* gene (NM_012330.4), showing the exon–intron organization (top), aligned with the corresponding MetaDome tolerance landscape and the multidomain structure of the encoded protein (bottom). Functional protein domains are color‐coded as indicated in the legend and include the NEMM (H15) domain, tandem PHD zinc‐finger domains (PHD1 and PHD2), the MYST‐type histone acetyltransferase (HAT) domain, the Asp/Glu‐rich acidic region, and the C‐terminal serine‐rich and methionine‐rich (SM) domains. Exons are color‐matched to the protein domains they encode. All pathogenic and likely pathogenic *KAT6B* variants identified through our literature review of molecularly characterized cases are mapped according to their genomic and protein positions. The two novel variants reported in the present study are highlighted in red. Variant labels are color‐coded according to the associated clinical phenotype: dark green, Say–Barber–Biesecker–Young–Simpson syndrome (SBBYSS); purple, Genitopatellar syndrome (GPS); pink, intermediate phenotype.

Analysis at the protein‐domain level provided additional insights. Variants affecting the catalytic HAT domain showed a striking enrichment for SBBYSS‐associated phenotypes. Among 19 reported variants involving this domain, all were associated with either SBBYSS or intermediate presentations, and no unequivocal GPS‐associated variants were identified (Table S1 and Figure 2). Although the number of observations remains limited, this finding raises the possibility that disruption of *KAT6B* acetyltransferase activity preferentially affects developmental pathways underlying the SBBYSS phenotype. In contrast, variants involving the acidic region displayed substantial phenotypic heterogeneity, being associated with GPS, SBBYSS, and intermediate phenotypes in relatively comparable proportions. Similarly, variants affecting the C‐terminal SM domain demonstrated a marked predominance of SBBYSS‐associated presentations, accounting for more than two‐thirds of reported cases (Table S1 and Figure 2). These observations parallel the functional organization of *KAT6B* and raise the possibility that disruption of catalytic versus transcriptional regulatory domains differentially influences developmental programs. Although this hypothesis requires functional validation, it provides a biologically plausible framework for interpreting the remarkable phenotypic diversity associated with *KAT6B* variants.

The novel c.5347G>T (p.Glu1783Ter) variant identified in Patient 1 further strengthens the association between distal exon 18/SM‐domain truncating variants and predominantly SBBYSS‐like phenotypes [10]. Beyond confirming this genotype–phenotype relationship, our patient expands the clinical characterization of this molecular subgroup by documenting severe dynamic upper airway dysfunction requiring respiratory support. These observations suggest that upper airway collapse may represent an intrinsic component of the SBBYSS phenotype rather than simply a secondary consequence of craniofacial dysmorphism.

The molecular findings observed in Patient 2 further expand both the mutational and mechanistic *spectrum* of KRDs. The novel de novo intronic variant c.3665‐21C>A, located outside the canonical splice acceptor sequence of intron 17, represents an uncommon class of pathogenic alterations whose functional interpretation remains particularly challenging. Clinically, the patient exhibited a severe multisystem phenotype largely overlapping with the GPS end of the *spectrum*, including agenesis of the CC, gyral simplification, severe intellectual disability, epilepsy, patellar agenesis, anorectal malformation, congenital hypothyroidism, renal abnormalities, hearing impairment, and significant upper airway dysfunction.

The availability of transcriptomic data provided a unique opportunity to investigate the functional consequences of this previously undescribed variant. Surprisingly, RNA sequencing performed on peripheral blood‐derived RNA demonstrated preservation of the canonical exon 17–18 splice junction, with no evidence of overt aberrant splicing involving the analyzed region. However, quantitative transcript analysis revealed an approximately two‐fold reduction in overall *KAT6B* expression compared with the unaffected mother, whereas housekeeping genes showed comparable expression levels between samples (Figure S1A,B). This pattern strongly suggests a gene‐specific effect rather than technical variability or global transcriptional differences. Although the precise molecular mechanism cannot be definitively established, the observed findings are compatible with preferential degradation of mutant transcripts, resulting in reduced abundance of functional *KAT6B* mRNA. Accordingly, our findings illustrate how transcript‐level quantitative abnormalities may constitute the primary functional evidence supporting pathogenicity even in the absence of overt splice‐junction alterations.

Beyond the characterization of a single variant, these observations have broader implications for the interpretation of noncanonical splice‐region variants. Increasing evidence from developmental disorders indicates that intronic variants may exert pathogenic effects through mechanisms that are not readily detected by conventional splice‐junction analyses. Reduced splicing efficiency, low‐abundance aberrant transcripts, allele‐specific transcript instability, or tissue‐specific splicing defects may all contribute to disease while remaining difficult to identify in peripheral blood‐derived RNA [19–21]. In this context, quantitative transcript abnormalities may represent a more informative functional readout than splice‐junction analysis alone. Our findings therefore support the integration of RNA‐based functional studies into the diagnostic evaluation of unresolved developmental disorders and may have broader relevance for interpreting the increasing number of extended splice‐region variants identified through exome sequencing.

Another clinically relevant finding emerging from our study concerns upper airway dysfunction. Although craniofacial abnormalities are well‐recognized features of KRDs, their functional consequences on airway dynamics have received comparatively limited attention. To our knowledge, only a few reports have provided detailed endoscopic characterization of upper airway abnormalities in affected individuals. Despite occupying opposite ends of the *KAT6B* phenotypic *spectrum*, both of our patients developed severe OSA requiring specialized respiratory management and exhibited remarkably similar endoscopic findings, including posterior displacement of the tongue base, retroglossoptosis, and secondary collapse of the epiglottis over the laryngeal vestibule. These shared features suggest that dynamic upper airway obstruction may represent a recurrent and underrecognized manifestation of KRDs rather than simply a secondary consequence of craniofacial dysmorphism. Considering the fundamental role of *KAT6B* in craniofacial morphogenesis and embryonic tissue patterning [6], it is biologically plausible that altered development of the mandible, palate, and upper airway collectively predisposes affected individuals to multilevel airway collapse. Systematic respiratory assessment, including sleep studies and endoscopic airway evaluation when clinically indicated, should therefore be considered as part of the routine multidisciplinary management of patients across the entire KRD *spectrum*.

Several limitations should be acknowledged. First, our study includes only two individuals, precluding definitive genotype–phenotype conclusions. Second, transcriptomic analyses were performed exclusively on peripheral blood‐derived RNA, which may not fully recapitulate transcriptional or splicing dynamics within disease‐relevant tissues, particularly the developing brain and craniofacial mesenchyme. Third, although the marked reduction in *KAT6B* expression strongly supports a functional effect of the c.3665‐21C>A variant, additional studies, including allele‐specific expression analyses, long‐read transcript sequencing, and disease‐specific cellular models, will be required to fully elucidate its molecular consequences. Finally, the literature‐derived genotype–phenotype analysis is inherently limited by reporting heterogeneity and variable clinical characterization across published cohorts. Nevertheless, the convergence of comprehensive clinical phenotyping, transcriptomic functional evidence, and an updated review of published molecularly confirmed KRDs provides complementary evidence supporting the pathogenic relevance of the identified variants while further refining current genotype–phenotype correlations across the *KAT6B* disease *spectrum*.

## 5. Conclusions

In conclusion, our study expands the molecular and clinical spectrum of KRDs through the description of two previously unreported de novo variants, including a noncanonical intronic variant supported by transcriptomic functional evidence. By integrating comprehensive clinical phenotyping with an updated review of molecularly characterized cases, we refine current genotype–phenotype correlations, provide additional evidence for domain‐specific phenotypic trends, and further support the concept of KRDs as a continuous developmental *spectrum* rather than discrete syndromic entities. Finally, our findings highlight the diagnostic value of RNA‐based analyses for the interpretation of noncanonical splice‐region variants and emphasize upper airway dysfunction as a clinically relevant but underrecognized manifestation that should be systematically considered during the multidisciplinary evaluation and follow‐up of affected individuals.

**Nomenclature**
ACMG: American College of Medical Genetics and Genomics
AMP: Association for Molecular Pathology
CADD: Combined Annotation Dependent Depletion
CC: corpus callosum
CNS: central nervous system
CPAP: continuous positive airway pressure
CT: computed tomography
GPS: Genitopatellar syndrome
HAT: histone acetyltransferase
KRDs: KAT6B‐related disorders
MRI: magnetic resonance imaging
NMD: nonsense‐mediated decay
OSA: obstructive sleep apnea
PEG: percutaneous endoscopic gastrostomy
PFO: patent foramen ovale
PHD: plant homeodomain
REVEL: Rare Exome Variant Ensemble Learner
SBBYSS: Say–Barber–Biesecker–Young–Simpson syndrome
SGA: small‐for‐gestational‐age
SM: serine/methionine–rich domain
WES: whole‐exome sequencing

## Author Contributions

Conceptualization: Vito Luigi Colona and Davide Vecchio; methodology: Maria Gnazzo, Alessandra Terracciano, Tommaso Mazza, and Antonio Novelli; formal analysis and investigation: Vito Luigi Colona and Maria Gnazzo; writing—original draft preparation: Vito Luigi Colona, Maria Gnazzo, Tommaso Mazza, and Alessandra Terracciano; writing—review and editing: Annalidia Donato, Lavinia Fioretti, Delia Monopoli, Paola Sabrina Buonuomo, Michaela Veronika Gonfiantini, Donatella Lettori, Roberta Taurisano, Daniela Concolino, Mafalda Mucciolo, and Marina Macchiaiolo; supervision: Antonio Novelli and Davide Vecchio.

## Funding

This study was supported by the Ministero della Salute (10.13039/501100003196).

## Disclosure

All authors have read and agreed to the submitted version of the manuscript.

## Ethics Statement

Ethical approval was not required for this study, as it involved the description of two clinical cases referred to our institution (Bambino Gesù Children′s Hospital, IRCCS, Rome, Italy) as part of routine clinical care. According to local regulations and institutional policies, ethics committee approval was therefore not required for this type of study. All biological samples and clinical data were collected after obtaining written informed consent from the patients′ legal guardians and in accordance with the principles of the Declaration of Helsinki. Written informed consent for publication of the clinical details and any accompanying images was obtained from the patients′ legal guardians. The submission of this manuscript was reviewed and approved by our Institution (n. RAP‐2026‐0002).

## Consent

Submission of the manuscript has been approved by our Institution (n. RAP‐2026‐0002).

## Conflicts of Interest

The authors declare no conflicts of interest.

## Supporting Information

Additional supporting information can be found online in the Supporting Information section.

## Supporting information


**Supporting Information 1** Figure S1: Splicing and expression analysis of the *KAT6B* gene. (A) Sashimi plot visualization of the *KAT6B* locus spanning exons 17 and 18. Read density tracks (histograms) and splice‐junction support (canonical arcs) demonstrate intact, preserved exon 17–18 splicing in both the proband and the mother. No aberrant splicing events, such as cryptic splice site activation, exon skipping, or loss of exon 18, are detectable in the proband sample. (B) Relative quantification of *KAT6B* mRNA expression levels in the proband and the mother. Expression values are normalized against housekeeping genes [*GAPDH* and *ACTB*], revealing a 2.1‐fold expression ratio (proband‐to‐mother).

**Supporting Information 2** Table S1: In silico analysis of *KAT6B* variants reported in the literature and those identified in the present study. For each variant, the corresponding exon/intron, domain, gnomAD frequency, ACMG classification, PMID, and associated phenotype are reported. Abbreviations: ACMG, American College of Medical Genetics and Genomics; C3, Class 3 (variant of uncertain significance); C4, Class 4 (likely pathogenic variant); C5 (pathogenic variant); CADD, Combined Annotation Dependent Depletion; N.A., not available; SBBYSS, Say–Barber–Biesecker–Young–Simpson syndrome; GPS, Genitopatellar syndrome.

## Data Availability

The data that support the findings of this study are available from the corresponding author upon reasonable request.
