# Supplementary material for: Clinical and Molecular Delineation of KAT6B‐Related Disorders: Novel Variants and Refined Genotype–Phenotype Correlations
Source: Hum Mutat. 2026 Sep 24;2026:6378376. doi: 10.1155/humu/6378376 (PMC13602169; doi:10.1155/humu/6378376)
Supplement: Supplementary file 1 [file HUMU-2026-6378376-s002.pdf]

A

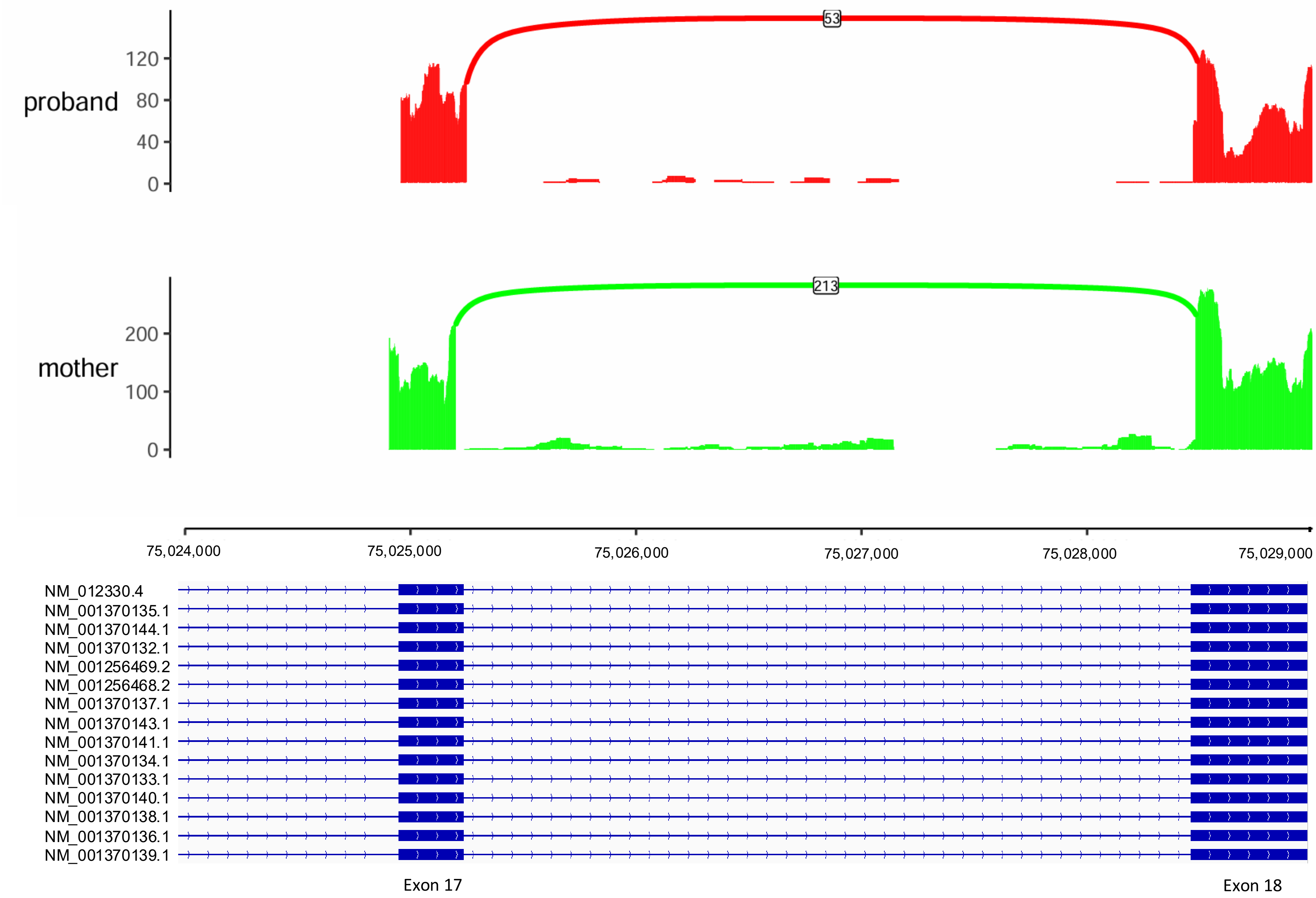
53
213
proband
mother
120
80
40
0
200
100
0
75,024,000
75,025,000
75,026,000
75,027,000
75,028,000
75,029,000
NM_012330.4
NM_001370135.1
NM_001370144.1
NM_001370132.1
NM_001256469.2
NM_001256468.2
NM_001370137.1
NM_001370143.1
NM_001370141.1
NM_001370134.1
NM_001370133.1
NM_001370140.1
NM_001370138.1
NM_001370136.1
NM_001370139.1
Exon 17
Exon 18

B

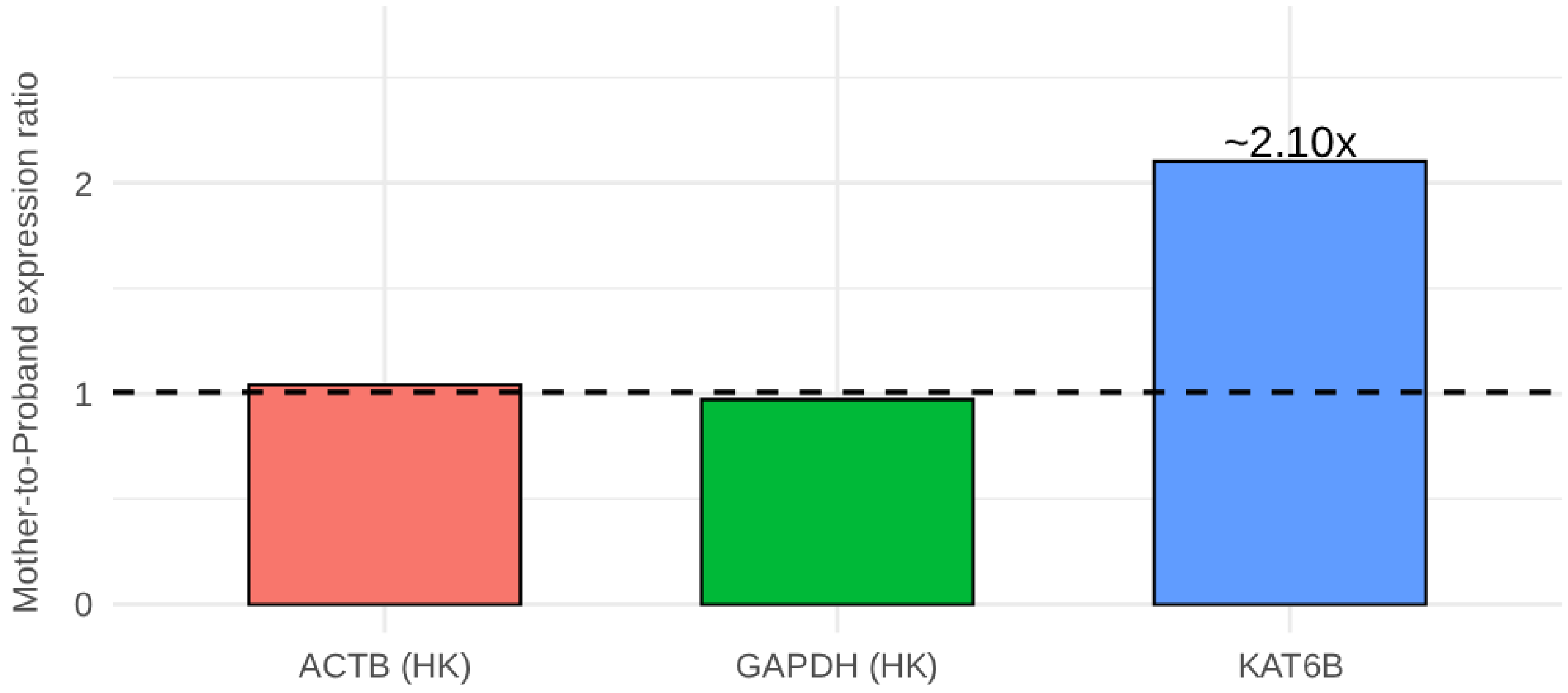
Mother-to-Proband expression ratio
0
1
2
~2.10x
ACTB (HK)
GAPDH (HK)
KAT6B
